# High failure rates of protease inhibitor-based antiretroviral treatment in rural Tanzania – A prospective cohort study

**DOI:** 10.1371/journal.pone.0227600

**Published:** 2020-01-13

**Authors:** Rahel E. Bircher, Alex J. Ntamatungiro, Tracy R. Glass, Dorcas Mnzava, Amina Nyuri, Herry Mapesi, Daniel H. Paris, Manuel Battegay, Thomas Klimkait, Maja Weisser, on behalf of the KIULARCO study group

**Affiliations:** 1 Ifakara Health Institute, Ifakara, Tanzania; 2 Swiss Tropical and Public Health Institute, Basel, Switzerland; 3 University of Basel, Basel, Switzerland; 4 Molecular Virology, Department Biomedicine Petersplatz, University of Basel, Basel, Switzerland; 5 St. Francis Referral Hospital, Ifakara, Tanzania; 6 Departments of Medicine and Clinical Research, Division of Infectious Diseases and Hospital Epidemiology, University Hospital Basel, Basel, Switzerland; CIRCB - Chantal BIYA International Reference Centre for Research on HIV/AIDS Prevention and Management, CAMEROON

## Abstract

**Background:**

Poor adherence to antiretroviral drugs and viral resistance are the main drivers of treatment failure in HIV-infected patients. In sub-Saharan Africa, avoidance of treatment failure on second-line protease inhibitor therapy is critical as treatment options are limited.

**Methods:**

In the prospective observational study of the Kilombero & Ulanga Antiretroviral Cohort in rural Tanzania, we assessed virologic failure (viral load ≥1,000 copies/mL) and drug resistance mutations in bio-banked plasma samples 6–12 months after initiation of a protease inhibitor-based treatment regimen. Additionally, viral load was measured before start of protease inhibitor, a second time between 1–5 years after start, and at suspected treatment failure in patients with available bio-banked samples. We performed resistance testing if viral load was ≥1000 copies/ml. Risk factors for virologic failure were analyzed using logistic regression.

**Results:**

In total, 252 patients were included; of those 56% were female and 21% children. Virologic failure occurred 6–12 months after the start of a protease inhibitor in 26/199 (13.1%) of adults and 7/53 of children (13.2%). The prevalence of virologic failure did not change over time. Nucleoside reverse transcriptase inhibitors drug resistance mutation testing performed at 6–12 months showed a positive signal in only 9/16 adults. No cases of resistance mutations for protease inhibitors were seen at this time. In samples taken between 1–5 years protease inhibitor resistance was demonstrated in 2/7 adults. In adult samples before protease inhibitor start, resistance to nucleoside reverse transcriptase inhibitors was detected in 30/41, and to non-nucleoside reverse-transcriptase inhibitors in 35/41 patients. In 15/16 pediatric samples, resistance to both drug classes but not for protease inhibitors was present.

**Conclusion:**

Our study confirms high early failure rates in adults and children treated with protease inhibitors, even in the absence of protease inhibitors resistance mutations, suggesting an urgent need for adherence support in this setting.

## Introduction

In Tanzania, as in many other sub-Saharan African (SSA) countries, there has been a tremendous increase in HIV care and treatment services over the past decade. This has reduced the prevalence of HIV infection to 4.6% [1]. Since the launch of free antiretroviral therapy (ART) in 2004 by the National AIDS Control Program, the number of individuals on therapy has increased from less than 5,000 people to one million in 2017 [2]. Although this is a positive development, there is an increasing incidence of treatment failures on first-line ART regimens—mostly with efavirenz or nevirapine combined with two nucleoside reverse transcriptase inhibitors (NRTI) [3]. A Tanzanian study from 2006–2009 showed a virologic failure (VF) rate of patients on first-line ART at 14.9% after a median of 26.1 months on therapy (interquartile range (IQR) 16.6–35.2). In all patients with virologic failure, 75.7% showed drug resistance mutations (DRM) to the backbone nucleoside analogues (NRTI) and to non-nucleoside reverse transcriptase inhibitors (NNRTI) [4]. In a previous study from our cohort in rural southern Tanzania, the overall VF rate was 9% in patients failing on first-line ART with 81% demonstrating DRM to NRTIs or NNRTIs [5].

Second-line treatment in Tanzania consists of a boosted protease inhibitor (bPI) combined with two NRTI. Additionally, in young children, a bPI-based treatment is currently started as a first-line therapy [3]. Children have a particularly high risk of virologic failure [6, 7], which puts them in jeopardy of having a lack of effective treatment options in the future. Thus far, several studies from SSA found that poor adherence rather than viral resistance is the main driver of failure under bPI treatment [8–10]. Information on DRM to bPI is crucial for future treatment guidelines, however only limited data is available from SSA.

In this study, we investigated the virologic outcome and development of DRM in HIV-1 infected adults and children on a bPI-containing regimen and identified risk factors for the development of treatment failure in a large rural HIV cohort in Tanzania.

## Materials and methods

### Study setting and participants

The Chronic Diseases Clinic at St. Francis Referral Hospital, Ifakara, Tanzania enrolls HIV-positive patients in a prospective cohort (Kilombero and Ulanga Antiretroviral Cohort (KIULARCO)). Written informed consent was obtained from the patient or, if younger than 18 years, the caregiver. Since its conception in 2005, KIULARCO enrolled more than 10,000 HIV-infected patients. Demographic, clinical, and treatment information is collected 4 times per year. Plasma is sampled twice yearly with storage in an onsite biobank. The cohort has been described in detail in other publications [11, 12]. For this study, we included all patients, enrolled into KIULARCO from 2005–2016, who were started on bPI-based ART, and who had a stored plasma sample taken 6–12 months after the start of treatment. We also used data from those newly enrolled on bPI treatment with a plasma sample taken at 6–12 months after enrolment. No routine viral monitoring was in place during the study period; however, the treating physician upon suspected immunologic or clinical failure could order viral load testing.

### Data collection

Data on demographics, clinical progression and ART was extracted from the KIULARCO electronic medical records. We recorded risk factors for treatment failure at the start of bPI treatment; namely sex, age, VF on first-line ART defined as viral load (VL) ≥1,000 copies/mL, advanced HIV disease (CD4 cells <200/μL and/or WHO stage III/IV), BMI (only in adults), and self-reported non-adherence [4, 13]. VL was tested on samples stored 6–12 months after bPI start (‘6–12 months sample’). If available, VL was additionally measured on samples taken within 6 months prior to start of bPI (‘pre-bPI sample’). Furthermore, we determined patients’ VL in a sample from >12 months after initiation of bPI, and at the time of clinical or immunologic treatment failure on bPI treatment (‘failure sample’). Resistance testing was carried out in samples with a VL ≥1,000 copies/mL, if amplification was successful.

### HIV quantification and analysis

Cell-free plasma was collected after centrifugation of EDTA blood at 1,900 rcf for 5 minutes and stored at -20°C until tested for VL, DRM or, after 2 weeks of being stored at -80°C. HIV RNA was extracted from 200 μL of plasma using the Pure Link Viral RNA/DNA Mini Kit (Thermofisher Scientific, Allschwil, Switzerland), according to the manufacturer’s instructions. Viral RNA quantification was performed with a validated in-house protocol with the Taqman qPCR Master Mix (Thermofisher) using the StepOne^TM^ System (Thermofisher), with a detection limit of 100 viral RNA copies/mL; details have been reported elsewhere (dx.doi.org/10.17504/protocols.io.749hqz6) [14]. For all VLs above 1000 copies/mL, genotyping of HIV-1 DRM was performed with a validated in-house protocol by population sequencing on an ABI 3130 capillary genetic analyzer, using six primers covering protease and reverse transcriptase [14]. HIV-1 drug resistance was interpreted according to the Stanford HIV Drug Resistance Database algorithm, version 8.4 (http://hivdb.stanford.edu). All DRM conferring high-level resistance were considered. CD4 cell counts were determined by flow cytometry (FACS Calibur, BD Company, Franklin Lakes, New Jersey, USA or Partec Cyflow counter, Partec GmbH, Germany).

### Outcomes

The primary outcome was VF, defined as HIV-1 RNA levels ≥1000 copies/mL. The secondary outcome was the prevalence of drug resistance in patients with VF, as per the Major HIV-1 DRM list [15].

### Definitions

Baseline was defined as the time point of starting a bPI ART or, if already on bPI ART, at inclusion into the cohort; variables and co-variates for risk factor analyses were considered up to 6 months before this date. Non-adherence was defined as missed at least one pill at any time during the past 6 months. VL results between 100–1000 copies/mL were defined as low-level viremia (LLV), whereas levels ≥1000 copies/mL where defined as VF. Advanced HIV disease was defined as CD4 cells <200/μL and/or a clinical WHO stage III/IV.

### Ethical considerations

The study was conducted according the current version of the World Medical Association Declaration of Helsinki. The KIULARCO study was approved by the Ifakara Health Institute institutional review board (IHI/IRB/No 16–2006), the Health Research Ethics Review Committee of the National Institute for Medical Research of Tanzania (NIMR/HQ/R.8a/Vol. IX/620), and the Ethical Review Board of the Canton of Basel, Switzerland.

### Statistical analyses

Demographic factors were summarized using frequencies and percentages for categorical data and medians and IQR for continuous data. Univariable and multivariable logistic regression models were used to estimate the association between risk factors and VF at 6–12 months. Non-linearity of the association with continuous variables was tested and modeled accordingly. Results are presented as odds ratios with 95% confidence intervals. Statistical analyses were performed using STATA 14 (StataCorpLP, College Station, TX, USA).

## Results

### Patient characteristics

**Fig 1:**
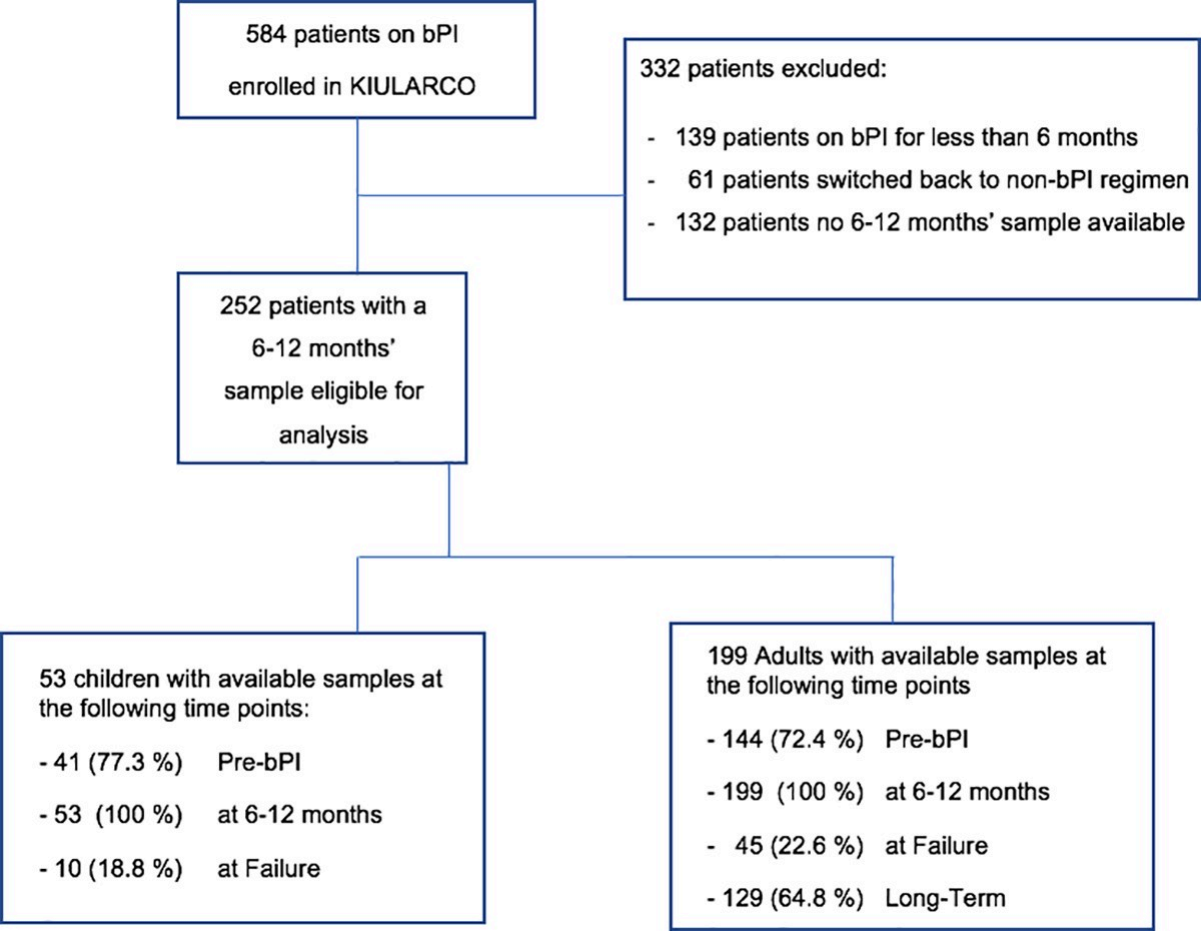
Overview of available samples from included patients. Profile of the study cohort on second-line at the Chronic Disease Clinic Ifakara in Ifakara, Morogoro, Tanzania. (KIULARCO Kilombero and Ulanga Antiretroviral Cohort; bPI boosted Protease Inhibitors; FUP follow-up).

Between 2005–2016, 584 participants of the KIULARCO cohort, received a bPI-based treatment (**Fig 1**). Of these, 332 were not eligible for the following reasons: being on bPI for a duration of less than 6 months, being switched back to a non-bPI regimen before 6 months, or no sample available 6–12 months after bPI start. Characteristics at the start of bPI treatment of the eligible 252 patients (199 adults and 53 children) included in the final analysis are summarized in **Table 1**. Out of 199 adults, 121 (60.8%) were female (2 were pregnant), median age and BMI were 41 years (IQR 33–49), and 21.5 (IQR 19.1–23.5) respectively. An advanced WHO stage (III/IV) was recorded in 105 (52.8%) patients. The median CD4 cell count was 163/μL (IQR 71–276), and median time on ART before start of a bPI was 3.6 years (IQR 1.5–5.8). Out of 53 children, 20 (37.7%) were female, median age was 8 years (IQR 5–10), 39 (73.6%) were in WHO stage III/IV, and median CD4 cell count was 590/μL (IQR 250.0–940.5) with a median time on first-line ART of 3.3 years (IQR 2–5.2).

**Table 1:** Patient’s characteristics at start of a boosted protease inhibitor^a^.

	Adults	Children	Overall
n	199	53	252
Female sex, n (%)	121 *(60*.*8)*	20 *(37*.*7)*	141 *(56*.*0)*
Age, median years (IQR)	41 *(33–49)*	8 *(5–10)*	36 *(16–46)*
Current pregnancy or delivery <3 months before start bPI, n (%)	2 *(1*.*0)*	-	2 *(0*.*8)*
Functional status, n (%)			
- Working	174 *(87*.*4)*	50 *(94*.*3)*	224 *(88*.*9)*
- Ambulatory	17 *(8*.*5)*	3 *(5*.*7)*	20 *(7*.*9)*
- Bedridden	1 *(0*.*5)*	*0 (0*.*0)*	1 *(0*.*4)*
BMI (kg/m^2^), median (IQR)	21.5 *(19*.*1–23*.*5)*	-	21.5 *(19*.*1–23*.*5)*
WHO clinical stage, n (%)			
- I/II	80 *(40*.*2)*	14 *(26*.*4)*	94 *(37*.*3)*
- III/IV	105 *(52*.*8)*	39 *(73*.*6)*	144 *(57*.*1)*
CD4 cell count/mm^3^, median (IQR)	163 *(71–276*.*0)*	590 *(250–940)*	205 *(84–364)*
- Missing values, n (%)	46 *(23*.*1)*	9 *(17*.*0)*	55 *(21*.*8)*
Years on 1^st^ line ART, median (IQR)	3.6 *(1*.*5–5*.*8)*	3.3 *(2–5*.*2)*	3.4 *(1*.*6–5*.*8)*
bPI based therapy at baseline	11 (5.5)	7 (13.2)	18 (7.1)
NRTI-backbone used during NNRTI-based ART^b^^,^^d^, n (%)			
- ZDV	133 *(66*.*8)*	41 *(77*.*4)*	174 *(69*.*0)*
- d4T	86 *(43*.*2)*	22 *(41*.*5)*	108 *(42*.*9)*
- TDF	78 *(39*.*2)*	4 *(7*.*5)*	82 *(32*.*5)*
Other (ddI, ABC)	3 *(1*.*5)*	4 *(7*.*5)*	7 *(2*.*8)*
NRTI-backbone used during bPI based ART^c^^,^^d^, n (%)			
- ZDV	63 *(31*.*7)*	9 *(17*.*0)*	72 *(28*.*6)*
- d4T	0 *(0*.*0)*	1 *(1*.*9)*	1 *(0*.*4)*
- TDF	166 *(83*.*4)*	38 *(71*.*7)*	204 *(81*.*0)*
- Other (ddI, ABC)	47 *(23*.*6)*	16 *(30*.*2)*	63 *(25*.*0)*
Reason for Start second-line, n (%)			
- Suspected Clinical Failure	20 *(10*.*1)*	0 *(0*.*0)*	20 *(7*.*9)*
- Suspected Immunologic Failure	112 *(56*.*3)*	15 *(28*.*3)*	127 *(50*.*4)*
- Suspected Virologic Failure	67 *(33*.*7)*	40 *(75*.*5)*	107 *(42*.*5)*

^a^ for patients registered on 2^nd^line treatment at enrolment

^b^ all 1^st^ line regimens contained 3TC/FTC additionally

^c^ 94% of 2^nd^ line regimens contained 3TC/FTC additionally

^d^ frequent switches in backbone drugs due to variable availability

IQR interquartile range, bPI boosted protease inhibitor, BMI body mass index, ART antiretroviral treatment, NNRTI Non-nucleoside reverse-transcriptase inhibitors, ZDV zidovudine, d4T stavudine, TDF tenofovir, ddI didanosine, ABC abacavir, 3TC lamivudine, FTC emtricitabine

The pre-bPI ART consisted of one NNRTI, mostly combined with zidovudine (69.9%), stavudine (42.9%) or tenofovir (32.9%). Due to reasons such as phasing out of certain regimens, adverse reactions, non-adherence, patient decision or stock-outs, many patients experienced several changes in their first-line treatment (median 2 (IQR 1–4)) before the start of a bPI-based treatment.

Eleven out of 199 (5.5%) adults and 7/53 (13.2%) children had already been prescribed a bPI treatment at the time of enrolment into our clinic. In adults, this was due to previously suspected treatment failure. In contrast, children were started directly on a bPI regimen, which was based on new guidelines [3]. At the start of the bPI-based regimen, tenofovir was the most commonly prescribed backbone drug (204 (81.0%)).

Reasons for prescribing a bPI-based regimen were immunologic failure in 127 patients (50.4%), clinical failure in 20 patients (7.9%), or as VL testing was not routine in Tanzania at the time of the study, suspected VF (107 patients (42.5%)).

### Virologic outcome and prevalence of DRM

#### Adults

VF was diagnosed after 6–12 months from initiation of bPI in 26/199 (13.1%) and LLV in 11/199 (5.5%) of adult patients (**Table 2**). In 16/26 (61.5%) patients with VF, DRM testing was done and revealed NRTI DRM in 9/16 (56.3%), NNRTI DRM in 14/16 (87.5%) and no bPI DRM. In 129 available samples taken between 1–5 years after start of bPI (>12 months on bPI), VF was diagnosed in 15 (11.6%) and LLV was present in 12 of the 129 patients (9.3%) at a median of 753 days (IQR 573–1033). Prevalence of VF and LLV were stable over this time period **(S1 Fig)**. DRM testing from samples taken between 1–5 years after bPI therapy was only available in 7/15 (46.7%) patients, revealing NNRTI DRM in all seven, while 4 had additionally NRTI DRM, and 2 had, beside NRTI and NNRTI DRM, also bPI DRM (**Table 2**).

**Table 2:** Virologic outcome of 656 samples in 252 patients at different time points.

	Pre-bPI -6-0 months	6–12 months	1–5 years	Failure on bPI
**Adults** (>15 years at start bPI)				
Available samples, n	144	199	129	45
- Virologic Failure, n *(%*)	60 *(41*.*7)*	26 *(13*.*1)*	15 *(11*.*6)*	11 *(24*.*4)*
- Low-Level viremia, n *(%)*	14 *(9*.*7)*	11 *(5*.*5)*	12 *(9*.*3)*	0 *(0*.*0)*
- Undetectable viral load, n *(%)*	70 *(48*.*6)*	162 *(81*.*4)*	102 *(79*.*1)*	34 *(75*.*6)*
DRM, n/tested isolates* *(%)*				
- NRTI	30/41*(73*.*2)*	9/16 *(56*.*3)*	4/7*(57*.*1)*	3/6*(50*.*0)*
- NNRTI	35/41*(85*.*4)*	14/16 *(87*.*5)*	7/7*(100*.*0)*	6/6*(100*.*0)*
- bPI	0/41*(0*.*0)*	0/16 *(0*.*0)*	2/7*(28*.*6)*	0/6 *(0*.*0)*
**Children** (<15 years at start bPI)				
Available samples, n	41	53	35	10
- Virologic Failure, n *(%)*	19 *(46*.*3)*	7 *(13*.*2)*	4 *(11*.*4)*	0 *(0*.*0)*
- Low-Level viremia, n *(%)*	6 *(14*.*6)*	4 *(7*.*5)*	3 *(8*.*6)*	2 *(20*.*0)*
- Undetectable viral load, n *(%)*	16 *(39*.*0)*	42 *(79*.*2)*	28 *(80*.*0)*	8 *(80*.*0)*
DRM, n/tested isolates* *(%)*				
- NRTI	15/16 *(93*.*8)*	0/1 *(0*.*0)*	1/3 *(33*.*3)*	0/0 *(0*.*0)*
- NNRTI	15/16 *(93*.*8)*	0/1 *(0*.*0)*	1/3 *(33*.*3)*	0/0 *(0*.*0)*
- bPI	0/16 *(0*.*0)*	0/1 *(0*.*0)*	0/3 *(0*.*0)*	0/0 *(0*.*0)*

bPI boosted protease inhibitor, DRM drug resistance mutation, NRTI Nucleoside Reverse Transcriptase Inhibitors, NNRTI Non-nucleoside reverse-transcriptase inhibitors

* number of strains with resistance out of successfully genotyped HIV DRM among patients with a VL = 1000c/mL

We analyzed 144 ‘pre-bPI samples’ to know whether there were pre-existing DRM. Of these, only 60 (41.7%) had confirmed VF and 14 (9.7%) had LLV. In the 41 sequences, available from samples before bPI start, 35 (85.4%) patients had NNRTI DRM and 30 patients (73.2%) had NRTI DRM. No bPI DRM was documented.

While on bPI treatment, 45 patients developed clinical or immunologic failure and had an additional sample stored. Of these, we found VF in 11 (24.4%) patients. Among the 6 available sequences all harbored NNRTI DRM, 3 harbored NRTI DRM, but no bPI DRM was observed. In patients with sequential samples available, mostly NNRTI and NRTI DRM were detected during all time points. Median time from start of bPI to diagnosed failure was 224 days (IQR 115–381).

#### Children

At 6–12 months, 7/53 (13.2%) children experienced VF and 4 (7.5%) had LLV. Sequencing was successful in only 1 out of 7 and no DRM were detected. In 35 children, samples taken between 1–5 years after bPI therapy (>12 months on bPI), showed that 4 (11.4%) had VF and 3 (8.6%) had LLV after a median of 581 days (IQR 467–743). Sequencing was successful in 3 of those, yielding 1 patient with NRTI and NNRTI DRM, but no bPI DRM.

In the 41 analyzed samples prior to therapy with bPI, 19 (46.3%) showed evidence of VF. After switching to bPI 16/19 re-suppressed, while 3 still had a VL >1000copies/mL. Two additional patients were suppressed before switch and experienced VF only in the 6–12 months sample. In the 16 pre-bPI samples with diagnosed VF, sequencing provided evidence of resistance with NRTI and NNRTI DRM in 15/16 (93.8%).

At the time of suspected clinical/immunologic failure on bPI, 2/10 (20.0%) had a positive VL in the range of LLV, but none had a documented VF. Median time to diagnosed failure was 93.5 days (IQR 82–179) in children.

### Prevalence of HIV subtypes

In our study population (85 available sequences from 75 patients) subtype C was most prevalent (35 isolates, 46.7%), followed by subtype A (23 isolates, 30.7%) and subtype D (9 isolates, 12%). This correlates well with the previously reported subtype distribution for the region [14]. Recombinant HIV-1 forms were identified in 8 samples, 7 of subtypes C and D, including CRF10_CD, and 1 of B/D.

### Mutational patterns of DRM

Overall, we found bPI DRM only in 2 adult patients. Both had been on a long-term bPI regimen (1027 and 1643 days, respectively) and both had accumulated multiple major bPI DRM. The first of these patients had the following mutations: *M46I*, *L76V*, *V82A* and *I84IV*. The second patient had *L24I*, *M46I*, *I50L*, *I54A*, and *V82A*.

The most common NNRTI mutation was *K103NS*, detected in 29 (51.8%) adults and 7 (36.8%) children. Additionally, *Y181CIV* was found in 15 (26.8%) and 4 (21.1%), *E138KAQG* in 10 (17.9%) and 4 (21.1%) and *G190ASEQ* in 12 (21.4%) and 5 (26.3%) adults and children, respectively.

For the NRTI, the most common non-TAM DRM was *M184VI*, which was found in 35 (62.5%) adults and 14 (73.7%) children. *TAM*s were found in 18 (32.1%) adults and 11 (57.9%) in children **(Table 3)**.

**Table 3:** Drug resistance mutations in patients with virologic failure.

*HIV drug resistance mutations*	Number of adults with resistance testing at all timepoints n = 56	Number of children with mutations n = 19
**NRTI-associated DRM n (%)**		
*M184VI*	35 (62.5)	14 (73.7)
*K65R*	9 (16.1)	0 (0.0)
*K70E*	0 (0.0)	1 (5.0)
*L74VI*	7 (12.5)	0 (0.0)
*Y115F*	2 (3.6)	0 (0.0)
*V75M*	4 (7.1)	0 (0.0)
*TAM*	18 (32.1)	11 (57.9)
**NNRTI-associated DRM n (%)**		
*L100I*	3 (5.4)	0 (0.0)
*K101PEH*	5 (8.9)	3 (15.8)
*K103NS*	29 (51.8)	7 (36.8)
*V106AM*	8 (14.3)	1 (5.3)
*E138KAQG*	10 (17.9)	4 (21.1)
*V179DEF*	2 (3.6)	3 (15.8)
*Y181CIV*	15 (26.8)	4 (21.1)
*Y188LCH*	6 (10.7)	0 (0.0)
*G190ASEQ*	12 (21.4)	5 (26.3)
*F227LC*	0 (0.0)	1 (5.3)
**bPI-associated DRM n (%)**		
*L24I*	1 (1.8)	0 (0.0)
*M46I*	2 (3.6)	0 (0.0)
*I50L*	1 (1.8)	0 (0.0)
*I54A*	1 (1.8)	0 (0.0)
*L76V*	1 (1.8)	0 (0.0)
*V82A*	2 (3.6)	0 (0.0)
*I84IV*	1 (1.8)	0 (0.0)

DRM drug resistance mutation, NRTI Nucleoside Reverse Transcriptase Inhibitors, NNRTI Non-nucleoside reverse-transcriptase inhibitors, bPI boosted protease inhibitor

### Adherence

Prior to starting bPI, 27/199 (13.5%) adults and 15/53 (28.3%) children had reported episodes of non-adherence to ART intake. After 6–12 months on bPI, 19/199 (9.5%) adults and 9/53 (17.0%) children still reported non-adherence **(S1 Table)**. Most common reasons for non-adherence were ‘having lost’ or ‘run out of’ medication, reported in 9/42 (21.4%) cases on first-line treatment and 11/28 (39.3%) on second-line treatment. One patient indicated depression as a reason for not taking pills during first-line treatment (1/42; 2.4%) and one on second-line (1/28; 3.6%). Reasons for poor adherence reported on first-line treatment only were ‘being too ill or too healthy’ (3/42; 7.1%), ‘being concerned about stigma’ (2/42; 4.8%) and ‘problems with delivery or stock out’ (1/42; 2.4%).

### Risk factors for failing on second-line

For adults, the presence of advanced HIV disease (WHO stages III/IV or CD4<200) was the only significant factor associated with a positive VL at 6–12 months on bPI (univariate analysis Odds ratio 5.04; 95% confidence interval 1.15–22.15, p = 0.03). For children, none of the investigated factors was significantly associated with a positive VL **(Table 4)**.

**Table 4:** Risk factor analysis for virologic failure at 6–12 months on boosted protease inhibitor.

	Univariable		Multivariable	
	OR (95% CI)	p-value	OR (95% CI)	p-value
**Adults**				
Male sex	1.39 (0.61–3.19)	0.44	1.97 (0.73–5.34)	0.18
Age	0.99 (0.95–1.02)	0.46	0.98 (0.94–1.03)	0.41
Advanced HIV disease	5.04 (1.15–22.15)	0.03	6.95 (0.89–54.22)	0.06
BMI	0.97 (0.87–1.08)	0.55	0.98 (0.86–1.10)	0.71
Non-Adherence on bPI	1.38 (0.37–5.12)	0.64	1.54 (0.37–6.41)	0.55
**Children**				
Male sex	4.23 (0.47–37.99)	0.20	3.06 (0.27–33.65)	0.36
Age	0.97 (0.80–1.20)	0.81	0.87 (0.62–1.21)	0.41
VF pre-bPI	1.87 (0.28–12.61)	0.51	1.94 (0.27–13.78)	0.51
Advanced HIV disease	2.12 (0.23–19.44)	0.51	0.85 (0.05–12.51)	0.91

OR odds ratio, CI confidence interval, BMI. Body mass index, bPI boosted protease inhibitor, VF virologic failure RNA levels ≥1000 copies/mL; advanced HIV disease: either CD4 cells <200/μL and/or WHO stage III/IV.

## Discussion

In this cohort from rural SSA we found a high rate of virologic failure on a bPI-based regimen despite a low emergence of resistance, which occurred exclusively after long-term drug exposure to protease inhibitors.

At 13%, the failure rate in adults and children, occurring early on bPI-based therapy, was higher than observed in the same cohort for patients on a long term first-line treatment, where resistance remained stable over time [5]. Similar studies on virologic failure rates on bPI-based treatment from other SSA settings report failure rates ranging from 11.1% [16] to 26% [17]. This trend was seen early after the start of bPI and remained stable over time. The high failure rates might be due to an inadvertent selection of patients with poor adherence. This hypothesis is supported by similar findings in other studies, which hardly found any viral resistance against bPI [18, 19]. In children from the same cohort, virologic failure rates on bPI were lower than the previously reported 25.4% on a first-line regimen [7]. A pediatric study from a similar cohort in Lesotho reported failure rates (VL ≥80 copies/mL) of 28% on first-line treatment and, despite counseling, of 49% after switching to bPI [6]. This data indicates key challenges in the management of HIV-infected children such as ART-acceptance and -tolerability, inability to swallow the pills mainly designed for adults, and likely pharmacokinetic issues [20].

The fact that HIV was suppressed on first-line treatment in about half of the patients with suspected treatment failure, supports the earlier notion that clinical deterioration or immunologic failure, based on the monitoring of CD4 cells and clinical condition alone, are poor predictors for VF [21, 22]. Possible reasons for an insufficient CD4 recovery other than an ongoing HIV replication could reside in vitamin and nutritional deficiencies, drug toxicities or alcohol intake. Interestingly, virologic failure rates were only 24.4% in adults with a diagnosis of clinical or immunologic failure on second-line treatment. This suggests that during second-line treatment, even more clinical situations were misclassified as “suspected treatment failures” than on first-line treatment, highlighting the need for routine viral monitoring, which was implemented in Tanzania shortly after completion of this study.

Side effects of bPI, e.g. diarrhea, are likely to lead to poor adherence and consequent ongoing virus replication, especially in those failing patients who had not previously failed on NNRTIs. Furthermore, it is possible that patients were sensitized to adherence questions and subsequently not admit to any poor adherence.

The high resistance rates for NNRTI in almost all virologically unsuppressed patients on first- or second-line treatment confirms that DRM do accumulate with time. This occurs when routine VL monitoring is not available and is a major concern with non-adherent patients, who are at risk of acquiring bPI DRM. The generally low bPI-related resistance rates can be attributed to the higher barrier to resistance development of this drug class [23] and the presumably lower time of exposure.

Our study confirms, in line with two other trials from SSA, that—even in the presence of NRTI resistance mutations—a triple drug regimen can retain efficacy with no obvious inferiority to other regimens [24, 25]. At the same time, our findings indicate that resistance through emerging mutated viruses is probably not the only cause for second-line treatment failure. However, this phenomenon is not fully understood.

It remains to be elucidated, whether resistance testing followed by guided treatment switching is beneficial, cost saving, and will reduce second-line treatment failures. Results of the ongoing REVAMP study in South Africa are anticipated to address this question [26]. Resistance data are urgently needed for defining optimal combinations of third-line drugs to be rolled out in Tanzania and other countries in SSA in the near future.

In our study, we measured LLV in 52 samples. In recent years, more literature on the significance of LLV is emerging [27, 28]. A study from Lesotho has shown a high percentage of resistance in these patients [29], challenging current guidelines, which classify only patients with a VL ≥1000 copies/mL as failing. Thus, a significant number of patients with lower VL might harbor resistance-associated mutations to the current regimen, increasing the risk for clinical failure and onwards transmission of resistance.

The only factor associated with VF at 6–12 months in adults was advanced HIV disease at the time of starting bPI. Factors such as an underlying untreated and progressing disease, associated weakness affecting the ability to travel and to attend clinical visits, drug intake, drug interactions, or side-effects of different medications leading to non-adherence could explain, why people with advanced illness more often had VF [13, 30].

Currently, the knowledge about second-line failure in Tanzania is scarce. The strength of this study is an even age distribution among those enrolled due inclusion of both children and adults. Another strength is the long follow-up time with corresponding samples available from different time points. Furthermore, this study adds resistance information, which is not available in many settings.

Our study has several limitations. The number of available samples of patients on bPI ART was relatively low. This was due to a significant variability in sample quality which led to inability to obtain sequencing results for every sample. This might have resulted in an underestimation of the true prevalence of viral drug resistance. Moreover, VL testing was done from bio-banked samples, and the definition of VF was based on a single VL, at the time neither confirmed by a second measurement nor followed by enhanced counseling, as recommended in the current national and WHO guidelines [3]. Lastly, adherence through self-reporting provides a rough estimate of pill intake. This practice in the absence of therapeutic drug monitoring may lead to underestimation of poor adherence. Moreover, patients on bPI have already failed on a first-line treatment, thus representing a selection of patients at higher risk for poor adherence.

In conclusion, we demonstrate that VF can occur early after the start of bPI, remaining stable over time and is best explained by poor adherence and less well indicated by overt drug resistance mutations. High early failure rates with overall poor adherence are worrisome as they may pave the way to further and accelerated resistance, clinical failure and higher rates of viral transmission especially of drug-resistant HIV strains. Therefore, better monitoring tools to improve adherence are urgently needed in rural settings.

## Supporting information

S1 Fig Long-Term outcomes after start of a boosted protease inhibitor. x-axis: years after start of bPI treatment
y-axis: % of patients
*after start bPI.
(TIF)

S1 Table Adherence. *Non-Adherence: Reported frequency of missing of ≥ 1 pill(s).
(DOCX)

S1 Dataset (XLSX)
