# Supplementary figures and images for: High failure rates of protease inhibitor-based antiretroviral treatment in rural Tanzania – A prospective cohort study
Source: PLoS One. 2020 Jan 13;15(1):e0227600. doi: 10.1371/journal.pone.0227600 (PMC6957142; doi:10.1371/journal.pone.0227600)

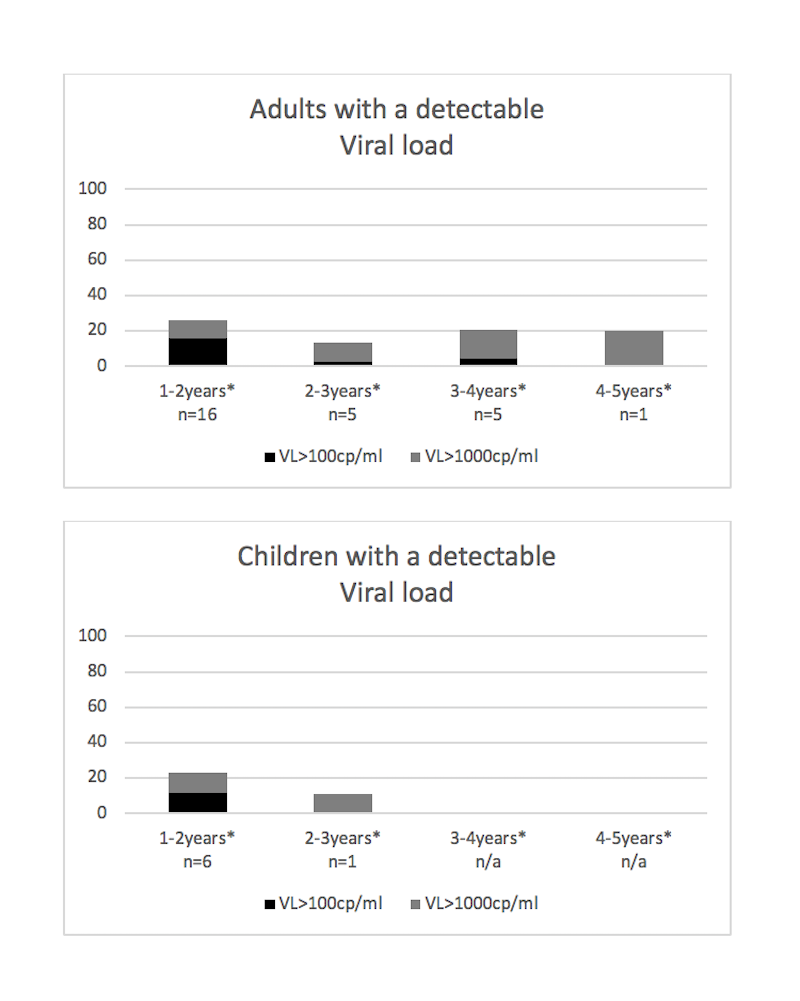

Supplement: S1 Fig — x-axis: years after start of bPI treatment y-axis: % of patients *after start bPI. (TIF) [file pone.0227600.s001.tif]
