# Supplementary material for: High failure rates of protease inhibitor-based antiretroviral treatment in rural Tanzania – A prospective cohort study
Source: PLoS One. 2020 Jan 13;15(1):e0227600. doi: 10.1371/journal.pone.0227600 (PMC6957142; doi:10.1371/journal.pone.0227600)
Supplement: S1 Table — *Non-Adherence: Reported frequency of missing of ≥ 1 pill(s). (DOCX) [file pone.0227600.s002.docx]

**Supplementary Table 1: Adherence**

|  | **Pre-bPI**  -6-0 months | | | **on bPI**  6-12 months | | |
| --- | --- | --- | --- | --- | --- | --- |
|  | Overall  252 | Adults  199 | Children  53 | Overall  252 | Adults  199 | Children  53 |
| **Non-Adherence*** | | | | | | |
| Never n (%) | 192 (76.2) | 161 (80.9) | 31 (58.5) | 215 (85.3) | 173 (86.9) | 42 (79.2) |
| Once a month n (%) | 21 (8.3) | 16 (8.0) | 5 (9.4) | 8 (3.2) | 6 (3.0) | 2 (3.8) |
| Twice a month n (%) | 3 (1.2) | 2 (1.0) | 1 (1.9) | 3 (1.2) | 2 (1.0) | 1 (1.9) |
| Once a week n (%) | 4 (1.6) | 0 (0.0) | 4 (7.5) | 4 (1.6) | 2 (1.0) | 2 (3.8) |
| Daily n (%) | 14 (5.6) | 9 (4.5) | 5 (9.4) | 13 (5.2) | 9 (4.5) | 4 (7.5) |
| n/a n (%) | 18 (7.1) | 11 (5.5) | 7 (13.2) | 9 (3.6) | 5 (2.5) | 2 (3.8) |

* Non-Adherence: Reported frequency of missing of ≥1 pill(s)
